# Structural basis for HCMV Pentamer recognition by neuropilin 2 and neutralizing antibodies

**DOI:** 10.1126/sciadv.abm2546

**Published:** 2022-03-11

**Authors:** Daniel Wrapp, Xiaohua Ye, Zhiqiang Ku, Hang Su, Harrison G. Jones, Nianshuang Wang, Akaash K. Mishra, Daniel C. Freed, Fengsheng Li, Aimin Tang, Leike Li, Dabbu Kumar Jaijyan, Hua Zhu, Dai Wang, Tong-Ming Fu, Ningyan Zhang, Zhiqiang An, Jason S. McLellan

**Affiliations:** 1 Department of Molecular Biosciences, The University of Texas at Austin, Austin, TX 78712, USA.; 2 Texas Therapeutics Institute, Brown Foundation Institute of Molecular Medicine, University of Texas Health Science Center at Houston, Houston, TX 77030, USA.; 3 Merck Research Laboratories, Merck & Co. Inc., Kenilworth, NJ 07033, USA.; 4 Department of Microbiology, Biochemistry and Molecular Genetics, Rutgers New Jersey Medical School, Newark, NJ 07103, USA.

## Abstract

Human cytomegalovirus (HCMV) encodes multiple surface glycoprotein complexes to infect a variety of cell types. The HCMV Pentamer, composed of gH, gL, UL128, UL130, and UL131A, enhances entry into epithelial, endothelial, and myeloid cells by interacting with the cell surface receptor neuropilin 2 (NRP2). Despite the critical nature of this interaction, the molecular determinants that govern NRP2 recognition remain unclear. Here, we describe the cryo-EM structure of NRP2 bound to Pentamer. The high-affinity interaction between these proteins is calcium dependent and differs from the canonical carboxyl-terminal arginine (CendR) binding that NRP2 typically uses. We also determine the structures of four neutralizing human antibodies bound to the HCMV Pentamer to define susceptible epitopes. Two of these antibodies compete with NRP2 binding, but the two most potent antibodies recognize a previously unidentified epitope that does not overlap the NRP2-binding site. Collectively, these findings provide a structural basis for HCMV tropism and antibody-mediated neutralization.

## Teaser

Cryo-EM structures reveal molecular basis of HCMV Pentamer recognition by NRP2 receptor and four neutralizing antibodies.

## INTRODUCTION

Human cytomegalovirus (HCMV) is a ubiquitous pathogen, with estimates of seropositivity ranging from 45 to 100% in different populations around the world (*1*). Although immunocompetent adults rarely experience any of the symptoms associated with HCMV infection, chronically immunocompromised individuals and recipients of solid organ transplants are susceptible to severe diseases including fatal hepatitis and encephalitis. In addition, congenital HCMV infection can cause debilitating and permanent birth defects (*1*–*3*). Despite the severity of these infections and the prevalence of this pathogen, there are currently no U.S. Food and Drug Administration–approved vaccines and therapeutic options remain limited (*4*–*6*).

HCMV is an enveloped, double-stranded DNA virus of the family Herpesviridae (*7*). The surface of the viral membrane is decorated by several glycoprotein complexes that mediate viral entry and membrane fusion (*8*–*10*). One of these complexes is the HCMV Trimer, composed of glycoproteins gH, gL, and gO (*11*, *12*). The HCMV Trimer mediates tropism for fibroblasts by binding platelet-derived growth factor receptor α (PDGFRα) (*13*, *14*). The HCMV Trimer is also capable of binding to transforming growth factor β receptor 3 (TGFβR3) with high affinity, suggesting that this interaction may mediate infection of a broader variety of cell types (*15*, *16*). The other critical tropism-determining complex is the HCMV Pentamer, which is composed of glycoproteins UL128, UL130, and UL131A and the same gH and gL proteins that comprise the bulk of the HCMV Trimer (*17*, *18*). This elongated heteropentamer mediates tropism for endothelial and epithelial cells by binding to neuropilin 2 (NRP2) and triggering the viral fusion protein, gB, to facilitate entry into host cells (*9*, *16*, *19*–*21*). It has been shown that continual passaging of HCMV in fibroblasts can lead to mutations in the pentamer-specific UL genes that disrupt the assembly of the Pentamer and prevent infection of both epithelial and endothelial cells (*9*, *22*–*24*), emphasizing the importance of Pentamer in the context of natural infection.

NRP1 and NRP2 are single-pass transmembrane proteins that are expressed on the surface of neuronal, epithelial, and endothelial cells (*25*, *26*). These proteins function as receptors and co-receptors that engage in numerous physiological processes, including angiogenesis and development of the nervous system (*27*, *28*). NRP2 is composed of two N-terminal CUB domains (a1 and a2), two F5/8 domains (b1 and b2), a MAM domain, a transmembrane domain, and a C-terminal PDZ domain that is thought to mediate cytoplasmic signaling in response to extracellular stimuli (*29*, *30*). Perhaps the most thoroughly characterized of these stimuli is vascular endothelial growth factor (VEGF) (*31*). The crystal structure of these proteins in complex with one another has been determined, revealing that the b1 domain of NRP2 engages the C-terminal arginine of VEGF (*32*, *33*). Since this initial characterization, the NRP2 b1 domain has been shown to interact with other binding partners via the same mechanism (*34*), prompting the moniker “CendR” to refer to this exposed C-terminal arginine motif (*35*). It has recently been shown that soluble NRP2 is capable of inhibiting HCMV infection of epithelial cells, and efforts to characterize this interaction have yielded a low-resolution negative-stain electron microscopy (EM) reconstruction of NRP2-bound Pentamer (*16*). Mass spectrometry of this chemically cross-linked sample has also provided some information about which residues are implicated in receptor binding. However, in the absence of high-resolution structural data, the molecular determinants that mediate this interaction remain unclear. Several additional Pentamer receptors have also been proposed, although the bases for these putative interactions have been less thoroughly characterized (*36*, *37*).

Previous efforts to characterize the humoral immune response to HCMV infection have yielded an extensive panel of neutralizing antibodies directed against gB, the Trimer, and the Pentamer (*38*–*40*). HCMV-directed antibodies isolated from asymptomatic donors predominantly target the Pentamer, suggesting that Pentamer-specific antibodies may restrict viral dissemination, making Pentamer a target for the development of vaccines and immunotherapeutics (*39*, *41*–*44*). On the basis of antibody binning and structural characterization, Pentamer has been divided into either seven antigenic sites that are largely confined to the membrane-distal UL apex (*16*, *41*), or four immunogenic regions (IRs) that span the entire surface of the Pentamer ectodomain (*38*). Correlation of neutralization potency to IR specificity has shown that antibodies directed against IR1, which encompasses the UL proteins, tend to be the most potently neutralizing, although these observations have been based on low-resolution negative-stain EM reconstructions and a relatively limited antibody repertoire (*38*).

To investigate NRP2 and antibody binding to the HCMV Pentamer, we initiated structural and biophysical studies. Here, we show that the interaction between Pentamer and NRP2 is calcium dependent and does not rely on the CendR-binding mechanism. By comparing the cryo-EM structures of Pentamer bound by either NRP2 or four neutralizing monoclonal antibodies (mAbs) isolated from memory B cells of HCMV-seropositive donors (*39*), we determine that the neutralization mechanism of two of these mAbs is NRP2 competition. We also show that the two most potently neutralizing mAbs recognize a previously undescribed antigenic site and do not compete with NRP2 binding, suggesting inhibition of additional events required for entry.

## RESULTS

### NRP2 binding to Pentamer is calcium dependent

**Fig. 1.**
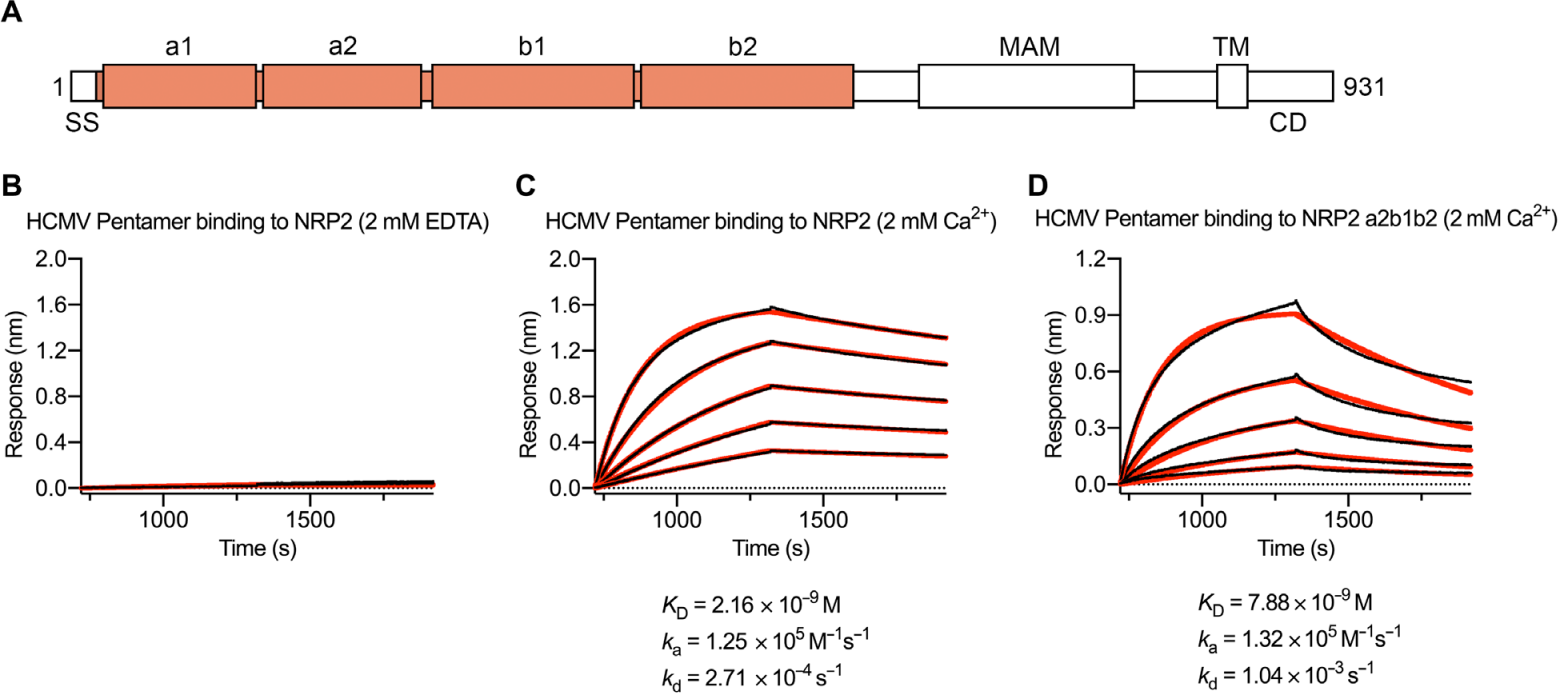
The interaction between NRP2 and Pentamer is calcium dependent. (**A**) Primary sequence diagram of NRP2. The construct that was used for structural studies is colored orange. SS, signal sequence; MAM, meprin, A-5 protein, and receptor protein-tyrosine phosphatase mu domain; TM, transmembrane domain; CD, cytoplasmic domain. (**B**) BLI sensorgram showing the absence of binding between Pentamer and NRP2 in the presence of 2 mM EDTA. (**C**) BLI sensorgram showing binding between Pentamer and NRP2 in the presence of 2 mM calcium. Data are shown as black lines, and best fit of a 1:1 binding model is shown as red lines. (**D**) BLI sensorgram showing binding between Pentamer and NRP2 a2b1b2 in the presence of 2 mM calcium. Data are shown as black lines, and best fit of a 1:1 binding model is shown as red lines.

**Fig. 2.**
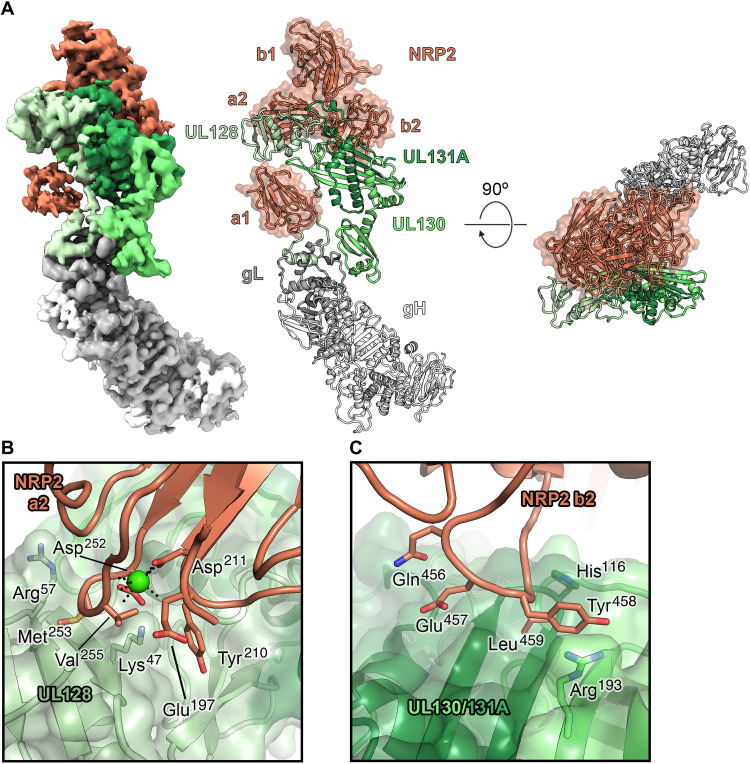
The cryo-EM structure of the HCMV Pentamer bound by NRP2. (**A**) Cryo-EM density is shown (left), with the Pentamer colored in shades of green, gray, and white and NRP2 colored orange. The atomic model of this complex (right) is shown as ribbons, with the NRP2 also represented by a transparent molecular surface. (**B**) Interface between the NRP2 a2 domain and UL128. UL128 is depicted as a transparent, green molecular surface with ribbons underneath, and NRP2 is shown as orange ribbons. Residues that are predicted to form critical contacts are shown as sticks. Oxygen, nitrogen, and sulfur atoms are colored red, blue, and yellow, respectively. The single calcium atom is shown as a bright green sphere, with black dotted lines depicting the interaction with conserved coordinating residues. (**C**) Interface between the NRP2 b2 domain and the HCMV Pentamer. ULs 130 and 131A are shown as a transparent, green molecular surface with ribbons underneath, and NRP2 is shown as orange ribbons, with residues predicted to form critical contacts shown as sticks. Oxygen and nitrogen atoms are colored red and blue, respectively.

On the basis of previous crystallographic experiments that reported conserved calcium-coordinating loops in both the a1 and a2 domains of NRP2 (Fig. 1A) (*45*, *46*), we measured the affinity of recombinantly expressed human NRP2 a1a2b1b2 for the soluble HCMV Pentamer ectodomain in both the presence and absence of calcium. We found that in the presence of 2 mM EDTA, no association between NRP2 and Pentamer could be detected. However, when the same experiment was performed in the presence of 2 mM CaCl_2_, the affinity of the interaction was determined to be 2.2 nM (Fig. 1, B and C), roughly 15-fold tighter than previously reported (*16*). This same NRP2 a1a2b1b2 construct was also capable of inhibiting HCMV infection of ARPE-19 cells in vitro (fig. S1). The addition of 2 mM CaCl_2_ enabled us to form a stable ~230-kDa complex that was suitable for cryo-EM screening. The addition of 0.1% amphipol A8-35 before deposition on EM grids helped to prevent aggregation and allowed for the determination of a 4.0-Å resolution cryo-EM structure of the HCMV Pentamer bound by human NRP2 (Fig. 2A and figs. S2 and S3A). Performing focused refinement on the NRP2-bound UL proteins yielded a 3.7-Å reconstruction that aided in model building and refinement.

### The cryo-EM structure of Pentamer bound to NRP2

These reconstructions revealed an extensive binding interface, with contacts formed by NRP2 domains a1, a2, and b2 (Fig. 2). Notably, the calcium-coordinating loop of domain a2 (residues 251 to 258) forms a sizable portion of this binding interface. Because Pentamer does not contribute to calcium coordination, we hypothesize that the calcium ion stabilizes the conformation of this NRP2 loop, thus allowing the interaction with Pentamer to occur (Fig. 2, A and B, and fig. S3D). Additional contacts are formed between the C-terminal β strands of ULs 130/131A and a loop formed by residues 453 to 461 of the b2 domain of NRP2 (Fig. 2C). This mode of NRP2 binding differs from the canonical CendR motif binding that has been described previously for other NRP2-binding partners (*47*, *48*). The CendR-binding mechanism involves the engagement of a C-terminal arginine residue by the b1 domain, whereas Pentamer is exclusively bound by the a1, a2, and b2 domains. Furthermore, none of the three UL proteins have an arginine as the C-terminal residue, which defines the CendR motif.

**Fig. 3.**
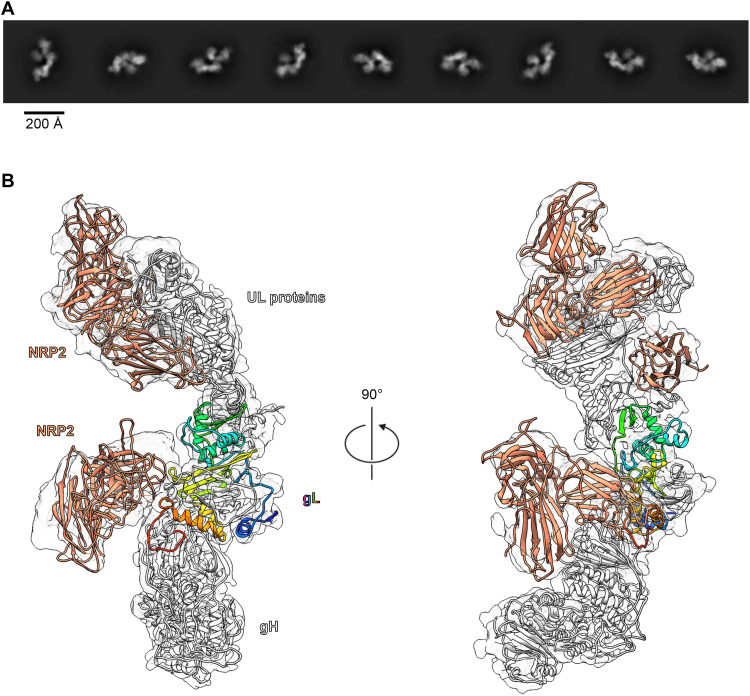
A subset of particles display a second copy of NRP2 bound to the C terminus of gL. (**A**) Two-dimensional class averages of Pentamer bound by two copies of NRP2. (**B**) A ~4.2-Å cryo-EM reconstruction of Pentamer bound by two copies of NRP2 is shown as a transparent surface. Atomic models of each component are docked in, shown as ribbons. Both copies of NRP2 are colored orange, and Pentamer is colored white, except for gL, which is colored as a rainbow blue-to-red from the N terminus to the C terminus. The a1 domain from the gL-bound copy of NRP2 was excluded because it could not clearly be resolved in this reconstruction.

As expected, most of the binding interface from the Pentamer is composed of the tropism-determining UL proteins, particularly UL128 and UL131A (*17*, *18*), which respectively contribute 437 and 208 Å^2^ of buried surface area to the interface. Whereas the NRP2 a2, b1, and b2 domains are clustered tightly together at the apex of the Pentamer, the N-terminal a1 domain is tethered via a flexible linker that allows it to bind near the middle of the Pentamer, where the C terminus of UL128 associates with gL. The local resolution for this portion of the reconstruction was relatively poor (~6.5 Å) compared to the rest of the complex, suggesting either conformational flexibility in this region or a loose association of the a1 domain. To test the importance of the a1 domain, we expressed NRP2 with a 144-residue N-terminal truncation. We observed that even in the absence of this flexibly tethered a1 domain, NRP2 a2b1b2 was capable of binding to the HCMV Pentamer with 7.9 nM affinity (Fig. 1D), supporting our structural observations that the critical determinants of Pentamer binding are contained within NRP2 domains a2b1b2. NRP2 a2b1b2 was also capable of effectively inhibiting HCMV infection of ARPE-19 cells in vitro (fig. S1). Intriguingly, our cryo-EM data processing also revealed that a second, more poorly resolved copy of NRP2 could be observed binding near the C-terminal arginine of gL via the b1 domain (Fig. 3 and fig. S2). Although this second NRP2 appears to exhibit the canonical CendR binding, it could only be observed in ~40% of particles. Furthermore, its binding to the gL protein rather than the tropism-determining UL proteins suggests that this second copy of NRP2 is likely an artifact of the high concentrations of NRP2 that were used to form a stable complex for EM studies. Overall, the conformation of the receptor-bound Pentamer ectodomain does not markedly differ from that of the unbound Pentamer (root mean square deviation of 2.2 Å over 333 Cα atoms) (fig. S4) (*49*). This observation suggests that rather than undergoing substantial conformational rearrangements, Pentamer acts as a tether to connect HCMV virions to the surface of epithelial and endothelial cells until gB fuses the viral and cellular membranes.

### The cryo-EM structures of Pentamer bound to four neutralizing human antibodies

**Fig. 4.**
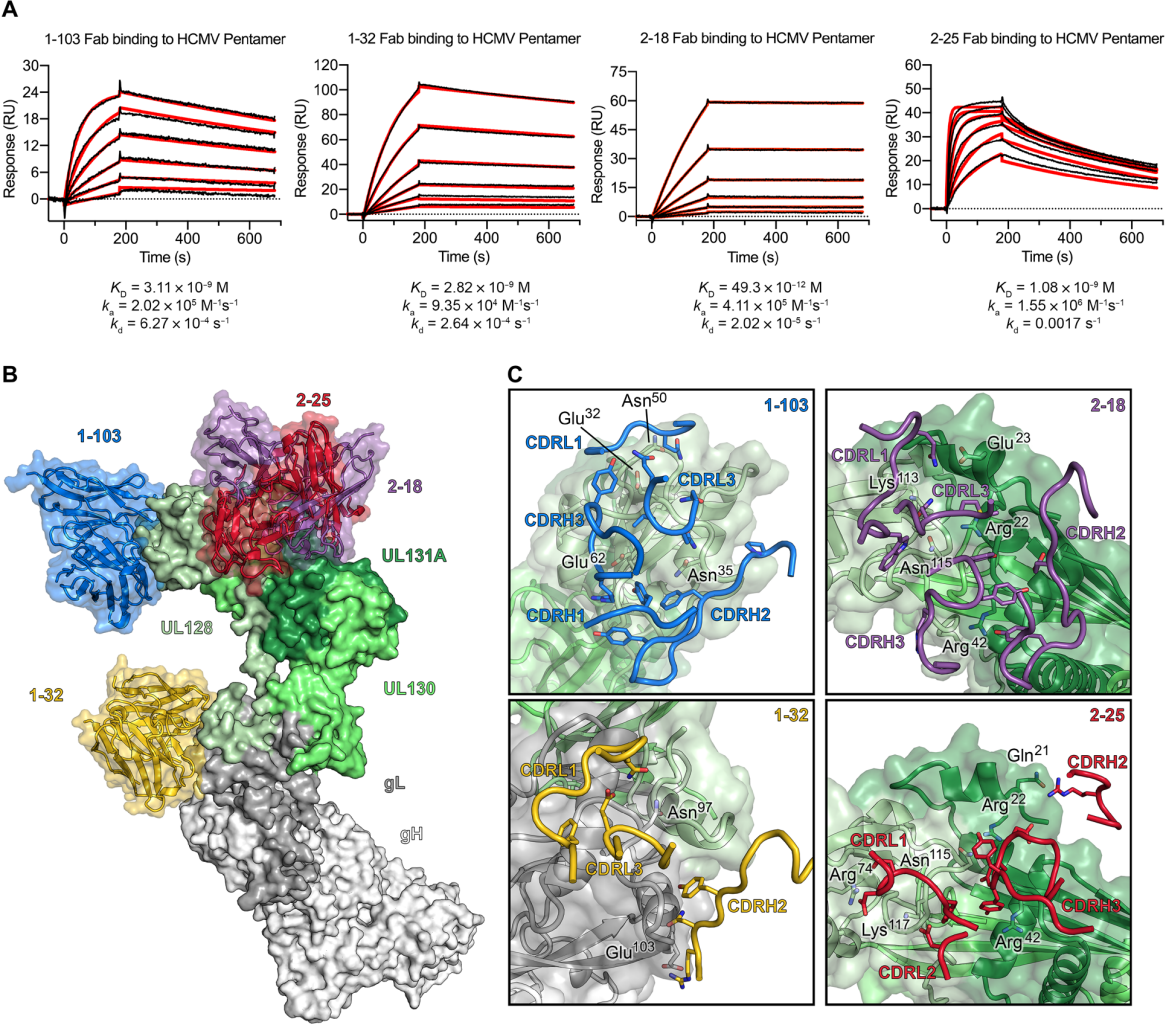
Composite of cryo-EM structures of Pentamer bound by four neutralizing human antibodies. (**A**) Surface plasmon resonance sensorgrams showing binding of each of the four neutralizing Fabs, with data shown as black lines and the best fit of a 1:1 binding model shown as red lines. (**B**) The atomic models of two cryo-EM structures of antibodies bound to the HCMV Pentamer are superimposed on the basis of the position of the UL proteins. The Pentamer is shown as a molecular surface, colored according to Fig. 2, and Fabs are shown as ribbons surrounded by a transparent molecular surface. Fab 1-103 is colored blue, Fab 1-32 is colored gold, Fab 2-18 is colored purple, and Fab 2-25 is colored red. (**C**) CDRs from each Fab are shown as ribbons, and the Pentamer is shown as a transparent molecular surface with ribbons underneath. Predicted critical contact residues are shown as sticks. Fab 1-103 (top left) is colored blue, Fab 1-32 (bottom left) is colored gold, Fab 2-18 (top right) is colored purple, and Fab 2-25 (bottom right) is colored red. Oxygen, nitrogen, and sulfur atoms are colored red, blue, and yellow, respectively.

To learn more about the mechanisms of neutralization of Pentamer-directed antibodies, we determined two cryo-EM structures of four naturally elicited human antibodies in complex with the Pentamer (Fig. 4 and figs. S3, S5, and S6). One complex was composed of Pentamer bound by 1-103, 1-32, and 2-25, and the other complex was composed of Pentamer bound by 2-18 and 8I21. The structure of 8I21 bound to Pentamer has been reported previously (*49*), and this Fab was added to aid in cryo-EM structure determination. The flexibility and elongated shape of the Pentamer necessitated focused refinements of the Fabs along with the domains making up their respective epitopes. Model building was facilitated by high-resolution crystal structures of unbound Fabs (1-103: 1.9 Å, 1-32: 2.1 Å, 2-18: 2.8 Å, and 2-25: 2.5 Å), which were then used as reference restraints and lightly refined as a part of the complex (tables S1 and S2). Three of these antibodies (1-103, 2-18, and 2-25) bound solely to the UL proteins at the head of the Pentamer, whereas the fourth (1-32) bound to gL near the junction between the UL proteins and the conserved gH/gL scaffold (Fig. 4B). The Fab 1-103 epitope is solely composed of residues from the membrane-distal tip of UL128, sometimes referred to as site 1 of IR1 (*16*, *38*). The epitopes of Fabs 2-18 and 2-25 overlap substantially with each other, with both Fabs binding to the junction between ULs 128 and 131A. This junction where UL128 meets UL131A does not fit into one of the preexisting antigenic sites, but rather overlaps with both sites 2 and 5 of IR1 (*16*, *38*), thus representing a previously undescribed neutralizing epitope. The Fab 1-32 epitope spans the interface between gH and gL, slightly below site 4/6 in IR2 (Fig. 4C) (*16*, *38*, *41*). This epitope is consistent with previous observations (*38*) that 1-32 is the only one of the four antibodies evaluated that is capable of binding to both the fully assembled HCMV Pentamer and disulfide-linked dimers of the gH/gL heterodimer. Despite the ability to recognize the gH/gL heterodimer that serves as the scaffold for assembly of both the HCMV Pentamer and Trimer, 1-32 was only capable of neutralizing HCMV infection in epithelial cells (*38*).

### Evaluating neutralization potency of Pentamer-directed antibodies

**Fig. 5.**
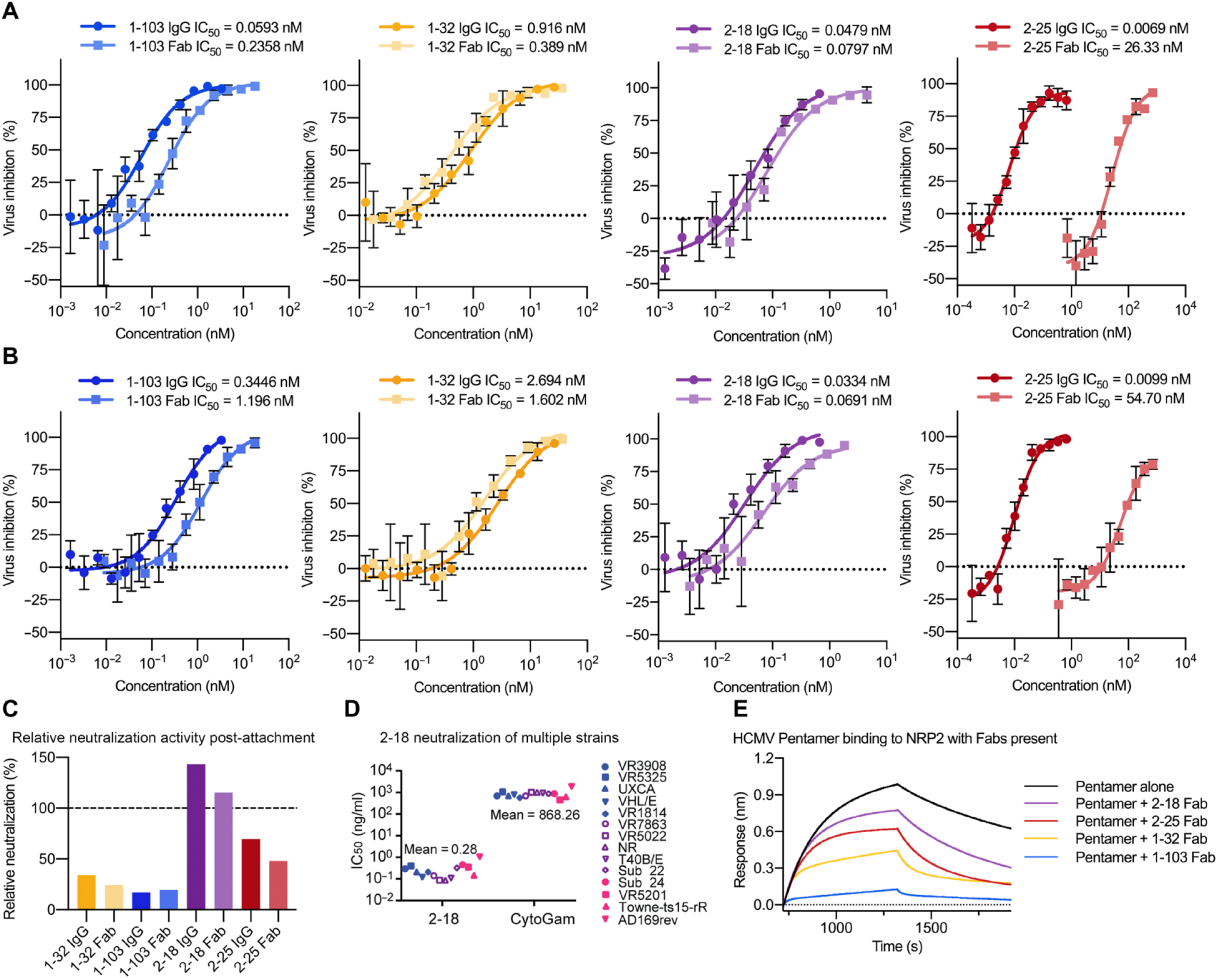
Antibodies 2-18 and 2-25 potently neutralize HCMV without disrupting NRP2 binding. (**A**) Neutralization curves are shown for each mAb based on inhibition of AD169rev-GFP infection in ARPE-19 cells. Inhibitory curves for both IgG and Fab are shown, with IgG shown in darker colors. (**B**) Post-attachment neutralization curves are shown for each mAb based on inhibition of AD169rev-GFP infection after adherence to ARPE-19 cells. (**C**) Relative IgG and Fab post-attachment neutralization potency. The reversed ratio of post-attachment IC_50_ to standard IC_50_ is plotted as a percentage for each IgG and Fab. (**D**) Neutralization potency of 2-18 IgG was evaluated against 12 clinical isolates and two laboratory-adapted HCMV strains in ARPE-19 cells. IC_50_ values were calculated by nonlinear fit of the percentage of viral inhibition versus concentration (ng/ml). The curves used to calculate IC_50_ values are shown in fig. S7. The neutralization results of mAbs 1-103, 1-32, and 2-25 against the same panel of HCMV strains have been reported previously (*38*). (**E**) Sensorgrams from a BLI-based competition experiment are displayed. NRP2 a1a2b1b2 was immobilized to a BLI sensor and dipped into Pentamer alone or Pentamer incubated with a molar excess of indicated Fab.

Neutralization of the four mAbs was evaluated using ARPE-19 cells and a laboratory-adapted strain of green fluorescent protein (GFP)–expressing HCMV with Pentamer restored (AD169rev-GFP). All four of these antibodies effectively neutralized HCMV when incubated with virions in a standard neutralization assay, with 2-18 exhibiting the most potent neutralization (Fig. 5A). Intriguingly, we observed a substantial decrease in the neutralization potency of 2-25 Fab, as compared to 2-25 immunoglobulin G (IgG). No such decrease in potency was observed for 2-18 Fab. The similarity in epitopes between these two mAbs implies that the decrease in 2-25 Fab potency can be attributed to the lower affinity of 2-25 compared to 2-18, suggesting that 2-25 requires the avidity effects of bivalency to neutralize effectively. We next performed a post-attachment neutralization assay by adding our mAbs to HCMV virions that had already adhered to ARPE-19 cells (Fig. 5B). Given that higher concentrations of antibody are typically required to inhibit adhered virions compared to free virions, it is expected that 1-103 and 1-32 exhibited substantially weaker neutralization potencies in this post-attachment assay. However, 2-18 and 2-25 were not susceptible to the same loss of potency, suggesting that they may neutralize via a mechanism distinct from that of 1-103 and 1-32 (Fig. 5C). 2-18 also retained its potent neutralizing activity when tested against a panel of 14 HCMV strains (Fig. 5D and fig. S7), as expected based on the high degree of sequence conservation in the UL proteins. On the basis of the differences in post-attachment neutralization potency that we observed, we next performed a biolayer interferometry–based competition assay to measure whether these Fabs were capable of disrupting the interaction between Pentamer and NRP2 (Fig. 5E). Consistent with the results of our post-attachment assay, 2-18 and 2-25 had a minimal effect on the interaction between NRP2 and Pentamer, whereas receptor binding was clearly disrupted in the presence of 1-103 and 1-32.

### Investigating mechanisms of antibody-mediated HCMV neutralization

**Fig. 6.**
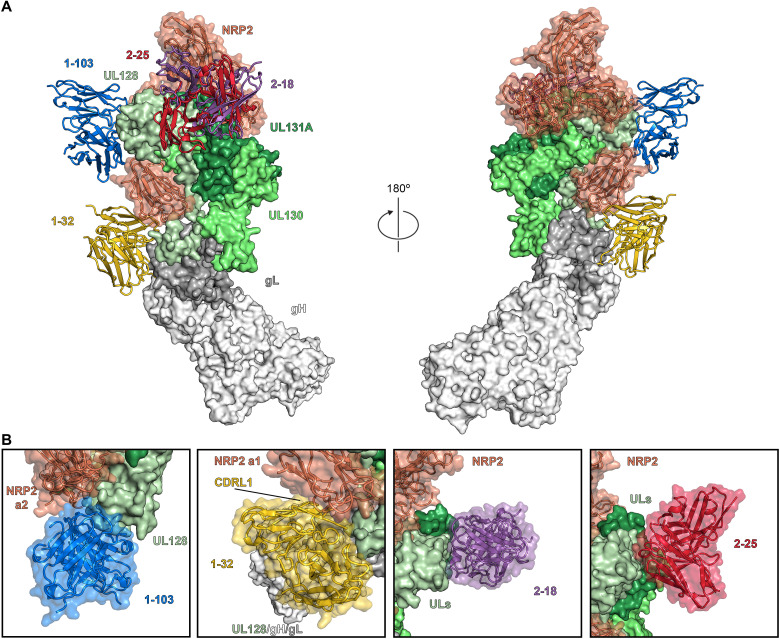
Pentamer-directed antibodies can neutralize HCMV via multiple mechanisms. (**A**) Cryo-EM structures of NRP2-bound Pentamer and Fab-bound Pentamer are superimposed on the basis of the position of the UL proteins. Pentamer is shown as a molecular surface colored according to Fig. 2, Fabs are shown as ribbons, colored according to Fig. 4, and NRP2 is shown as orange ribbons surrounded by a transparent molecular surface. (**B**) Close-up views of each Fab-bound Pentamer are superimposed upon the NRP2-bound Pentamer. Both Fabs and NRP2 are shown as ribbons surrounded by a transparent molecular surface, while the Pentamer is shown as a solid molecular surface. NRP2 is colored orange, 1-103 is colored blue, 1-32 is colored gold, 2-18 is colored purple, and 2-25 is colored red.

By analyzing the structures of these immunocomplexes in conjunction with the structure of Pentamer bound by NRP2, it becomes possible to delineate more clearly the molecular basis for neutralization (Fig. 6). The heavy chain complementarity-determining region 3 (CDRH3) and light chain complementarity-determining region 1 (CDRL1) of Fab 1-103 compete with NRP2 for binding to the same portion of Pentamer that is engaged by the calcium-coordinating loop (residues 251 to 258) of NRP2 domain a2. In contrast, Fab 1-32, which binds to the junction between gL and the UL head of Pentamer, occupies the same space as several of the loops of the a1 domain of NRP2 (residues 45 to 48; 72 to 77; 106 to 110) via its CDRL1 (Fig. 6B). Consistent with the results of our biolayer interferometry (BLI) assay and the relatively poor map for domain a1, we found that when bound by 1-32, Pentamer was still capable of interacting with NRP2, albeit with diminished affinity (133 nM) (fig. S8), further supporting our findings that the a1 domain is not strictly required for binding to Pentamer. This observation is also consistent with the relatively weak neutralization capacity of 1-32 compared to 1-103 (Fig. 5A), which competes for the a2b1b2 interface.

As expected based on the results of our NRP2 competition assay, the two most potently neutralizing mAbs that we examined, 2-18 and 2-25 (Fig. 5, A to C and E), do not appear to compete with NRP2 a1a2b1b2 based on our structural analysis. Although it is possible that 2-18 and 2-25 compete with the C-terminal MAM domain, this seems unlikely based on the position of the C terminus of b2 (*50*). These mAbs both bind to the junction between ULs 128 and 131A, and although this epitope is directly adjacent to the NRP2-binding interface, the binding angles of 2-18 and 2-25 result in these two Fabs being directed away from the β sheet–rich face of the Pentamer that is responsible for engaging NRP2. This broad and potent neutralization of multiple HCMV strains (Fig. 5D and fig. S7), which occurs without disrupting the interaction between NRP2 a1a2b1b2 and the Pentamer, suggests that 2-18 and 2-25 neutralize via a mechanism distinct from 1-103 and 1-32 (fig. S9). Whether 2-18 and 2-25 prevent the association of a secondary receptor or prevent some alternative signaling event that is required to trigger gB-induced membrane fusion remains unclear and requires additional investigation.

## DISCUSSION

Collectively, these data provide a molecular basis of HCMV tropism for both epithelial and endothelial cells, representing a critical advance in our understanding of how HCMV engages host cells at one of the earliest stages of infection (*20*, *51*). Similarly, the structure of the HCMV Trimer was recently reported in complex with PDGFRα and TGFβR3, two host cell receptors that both mediate tropism for fibroblasts (*13*, *15*). In an effort to explain how this interaction might lead to triggering of gB and viral fusion, the authors speculated that receptor engagement of the Trimer may induce a rigid-body rotation relative to the viral membrane that causes the attachment complex to dissociate from gB, thereby destabilizing the prefusion conformation and triggering fusion (*52*, *53*). Our findings with NRP2 and Pentamer agree with their observation that receptor binding does not induce substantial conformational changes within the gH/gL complex, lending credence to the hypothesis that a rigid-body rotation of the receptor binding complex may act as a trigger to induce membrane fusion. In this model, the association between Pentamer and gB on the viral surface may act like the pin in a grenade, preventing the metastable prefusion conformation from prematurely transitioning to the postfusion conformation. NRP2 binding to Pentamer would then hypothetically induce a rigid-body motion that causes Pentamer and gB to dissociate. However, the existence of neutralizing antibodies, such as 2-18 and 2-25, that do not disrupt NRP2 binding suggests that additional fusion triggers, perhaps in the form of secondary receptors (*36*, *37*), may exist, necessitating further investigation. Several additional HCMV receptors have been suggested, including OR14I1 (*36*), CD147 (*37*), and THBD, of which the latter has previously been shown to bind Pentamer with nanomolar affinity (*16*).

Here, we demonstrate that the high-affinity interaction between NRP2 and Pentamer requires calcium. Ion coordination by the NRP2 a2 domain, in particular, plays a crucial role in rigidifying the loop such that it is positioned to interact with the UL proteins. Rather than acting as a regulatory mechanism, it is more likely that this interaction has simply evolved to occur in the extracellular milieu, where calcium is constantly present at a concentration of ~1 mM (*54*). Although the homologous NRP1 shares this a2 calcium-coordinating loop, NRP1 also contains several short insertions in the Pentamer-interacting loops of the a2 domain (*45*). On the basis of our structural analysis, it appears that these insertions would cause steric clashes with the UL proteins, likely explaining the lack of binding between NRP1 and Pentamer (*16*). Previous negative-stain EM studies of the Pentamer bound to NRP2 appear to have been hampered by orientation bias of this elongated complex, although they largely agree with the cryo-EM data presented here (*16*). Mass spectrometry analysis of the chemically cross-linked sample correctly determined the importance of several critical contact residues that we identify in our cryo-EM structure, particularly Lys^47^ and Lys^92^ in UL128 (fig. S10A). Similarly, our cryo-EM structure agrees well with the results from charge cluster-to-alanine scanning previously performed on UL128 and UL131A (fig. S10B). Several of the identified charge clusters in UL128 and UL131A that disrupted endothelial cell infection are located at or near the NRP2 interface. However, charge clusters identified in UL130 were not located at or near NRP2 interfaces in our structure, suggesting that their effect on endothelial cell infection is due to disruption of other function(s) besides NRP2 binding (*55*–*57*).

In addition to detailing the molecular determinants that mediate HCMV infection, these findings expand our understanding of how antibody-mediated neutralization of HCMV can be achieved. Pentamer is indispensable for HCMV infection of epithelial and endothelial cells and facilitates viral dissemination that occurs in vivo through both cell-to-cell spreading and viral transfer to leukocytes (*19*, *58*). Many in vitro studies have shown that Pentamer-directed neutralizing antibodies are capable of preventing the infection of specific cell types that are implicated in HCMV dissemination (*59*–*62*). Furthermore, an early and robust antibody response to Pentamer is associated with a significantly reduced risk of fetal HCMV transmission (*63*). Although Pentamer-directed neutralizing antibodies that do not target the UL proteins have been reported (*15*, *64*), the most potently neutralizing antibodies recognize the ULs that make up IR1 (*38*). The data reported here show that antibodies such as 1-103 and 2-18 neutralize with different mechanisms, although they are directed against the same IR. Our findings suggest that it may be possible to neutralize HCMV via multiple distinct mechanisms simultaneously by administering a cocktail of Pentamer-directed antibodies (*44*, *60*, *65*). By elucidating which epitopes on the surface of the Pentamer are most susceptible to antibody-mediated neutralization, these findings will also help to guide the development of therapeutic interventions that might be administered prophylactically to prevent transplant-induced reactivation or congenital transmission.

During the preparation of this manuscript, it came to our attention that our colleagues Kschonsak *et al.* had performed similar structural studies of the HCMV Pentamer bound to NRP2 and our respective manuscripts were co-submitted for review. Although they do not observe a single pentamer bound by two copies of NRP2, the findings of Kschonsak *et al.* closely agree with the data presented here, but they also report the observation of a complex composed of two copies of Pentamer, which appear to have dimerized after binding a single copy of NRP2. These data are particularly intriguing when considering the putative neutralization mechanism of 2-18– and 2-25–like antibodies. Because the dimerization interface is formed by two copies of UL128, it is likely that the binding of 2-18 and 2-25 would prevent dimerization from taking place while still allowing NRP2 engagement to occur. It is likely that 1-32 would also interfere with Pentamer dimerization; however, this antibody also competes with NRP2 domain a1, as we show here. Although this phenomenon warrants further investigation, these findings suggest that Pentamer dimerization after receptor engagement may represent a critical step in the process of HCMV entry that can be targeted and prevented by mAbs such as 2-18 and 2-25.

## METHODS

### Protein production and purification

Plasmids encoding the heavy and light chains of 1-103, 1-32, 2-18, 2-25, and 8I21 IgG with an HRV3C protease cleavage site engineered into the hinge between the CH1 and CH2 domains of the heavy chain were cotransfected into FreeStyle 293F cells using polyethylenimine. To produce the soluble ectodomain of the HCMV Pentamer (strain Towne), plasmids encoding residues 24 to 718 of gH with a C-terminal 6× HisTag, residues 31 to 278 of gL, residues 21 to 171 of UL128, residues 26 to 214 of UL130, and residues 19 to 129 of UL131A, all with artificial signal sequences, were simultaneously cotransfected at an equimolar ratio.

Similarly, plasmids encoding an artificial signal peptide, residues 23 to 595 of human NRP2, and a C-terminal HRV3C cleavage site with either an 8× HisTag and a TwinStrepTag or a monomeric IgG1 Fc tag and an 8× HisTag were transfected into FreeStyle 293F cells, as described above. An N-terminal truncation of NRP2 that encompassed residues 145 to 595 with an artificial signal sequence and a C-terminal HRV3C cleavage site with a monomeric IgG1 Fc tag and an 8× HisTag (NRP2 a2b1b2) was transfected using the same conditions. NRP2 and NRP2 a2b1b2 were purified from cell supernatants using either StrepTactin resin (IBA) or Protein A resin before being run over a Superdex 200 Increase column using a buffer composed of 2 mM tris (pH 8.0), 200 mM NaCl, 0.02% NaN_3_, and 2 mM CaCl_2_.

To form the complex of Pentamer + 1-103 + 1-32 + 2-25, purified 1-103 IgG was immobilized to Protein A resin and this 1-103 resin was then used to capture Pentamer from cotransfected cell supernatants. The 1-103 + Pentamer complex was then eluted by incubation with HRV3C protease and purified over a Superose 6 Increase column in 2 mM tris (pH 8.0), 200 mM NaCl, and 0.02% NaN_3_. This complex was then passed over a column containing 2-25 IgG immobilized to Protein A resin. Again, the complex was eluted by incubation with HRV3C protease, and a molar excess of 1-32 Fab was added before a final round of purification over a Superose 6 Increase column using the same buffer.

To form the Pentamer + 2-18 + 8I21 complex, purified 2-18 IgG was immobilized to Protein A resin and used to capture Pentamer from cotransfected cell supernatants. The 2-18 + Pentamer complex was then eluted by incubation with HRV3C protease and mixed with a molar excess of 8I21 Fab before being run over a Superose 6 Increase column in 2 mM tris (pH 8.0), 200 mM NaCl, and 0.02% NaN_3_.

To form the Pentamer + NRP2 complex, purified Pentamer was mixed with a threefold molar excess of 8× His/TwinStrep-tagged NRP2 in a buffer composed of 2 mM tris (pH 8.0), 200 mM NaCl, 0.02% NaN_3_, and 2 mM CaCl_2_, and the two components were allowed to bind on ice for 1 hour. This mixture was then purified over a Superose 6 Increase column (Cytiva) using the same buffer.

### X-ray crystallographic studies

Purified IgGs 1-103, 1-32, 2-18, and 2-25 were incubated with 10% (w/w) His-tagged HRV3C protease on ice for 2 hours before being passed over Protein A and Ni-NTA resin to remove cleaved Fc and excess protease. The remaining Fab was purified by size exclusion chromatography using a Superdex 200 Increase column in 2 mM tris (pH 8.0), 200 mM NaCl, and 0.02% NaN_3_ (1-132 and 2-18) or 2 mM tris (pH 8.0), 50 mM NaCl, and 0.02% NaN_3_ (1-103 and 2-25).

1-103 Fab was concentrated to 15.0 mg/ml and used to prepare sitting-drop crystallization trays. Diffraction-quality crystals grew in a mother liquor composed of 2.1 M sodium formate, 25% polyethylene glycol (PEG) 3350, 0.1 M sodium acetate (pH 4.5), and 0.1 M calcium chloride. 1-103 Fab crystals were cryoprotected using mother liquor supplemented with 20% glycerol before being plunge-frozen into liquid nitrogen.

1-32 Fab was concentrated to 11.0 mg/ml and used to prepare hanging-drop crystallization trays. Diffraction-quality crystals were grown in 2.0 M ammonium sulfate, 0.2 M sodium chloride, and 5% isopropanol. 1-32 Fab crystals were cryoprotected using mother liquor supplemented with 20% glycerol before being plunge-frozen into liquid nitrogen.

2-18 Fab was concentrated to 12.0 mg/ml and used to prepare sitting-drop crystallization trays. Small crystalline needles, grown in 0.1 M Hepes (pH 7.5), and 45% PEG 400 were used to perform microseed matrix screening, ultimately yielding diffraction-quality crystals in a mother liquor composed of 0.2 M ammonium acetate, 0.1 M sodium citrate tribasic dihydrate (pH 5.6), and 30% PEG 4000. 2-18 Fab crystals were cryoprotected using mother liquor supplemented with 20% glycerol before being plunge-frozen into liquid nitrogen.

2-25 Fab was concentrated to 15.4 mg/ml and used to prepare sitting-drop crystallization trays. Diffraction-quality crystals were grown in 30% PEG 4000 and a mixture of 0.2 M divalent cations (*66*) and 0.1 M bis-tris (pH 6.5). 2-25 Fab crystals were looped without cryoprotectant and directly plunge-frozen into liquid nitrogen.

All diffraction data were collected at Argonne National Laboratory, Advanced Photon Source, SBC-19ID. Datasets were indexed in iMOSFLM (*67*) and scaled in AIMLESS (*68*). Molecular replacement solutions were determined using PhaserMR (*69*), and models were iteratively built and refined using Coot (*70*), PHENIX (*71*), and ISOLDE (*72*). Full crystallographic data collection and refinement statistics can be found in table S1. Crystallographic software packages were curated by SBGrid (*73*).

### Cryo-EM sample preparation and data collection

Purified HCMV Pentamer + 2-18 + 8I21 complex was diluted to a concentration of 0.25 mg/ml in 2 mM tris (pH 8.0), 200 mM NaCl, 0.02% NaN_3_, and 0.01% amphipol A8-35. 8I21 Fab was added after initial attempts to visualize the Pentamer + 2-18 complex were hampered by a lack of distinguishable features. The ternary complex (3 μl) was deposited on a CF-1.2/1.3 grid that was glow-discharged at 25 mA for 1 min using Emitech K100X (Quorum Technologies). Excess liquid was blotted away for 6 s in a Vitrobot Mark IV (FEI) operating at 4°C and 100% humidity before being plunge-frozen into liquid ethane. Data were collected on a Titan Krios (FEI) operating at 300 kV, equipped with a K3 direct electron detector (Gatan). Movies were collected using Leginon (*74*) at a magnification of ×22,500, corresponding to a pixel size of 1.047 Å.

Purified HCMV Pentamer + 1-103 + 1-32 + 2-25 complex was diluted to a concentration of 0.2 mg/ml in 2 mM tris (pH 8.0), 400 mM NaCl, 0.02% NaN_3_, and 0.01% amphipol A8-35. Protein (3 μl) was deposited on a CF-1.2/1.3 grid that was plasma-cleaned at 25 mA for 30 s using a Solarus plasma cleaner (Gatan). Excess liquid was blotted away for 6 s in a Vitrobot Mark IV (FEI) operating at 4°C and 100% humidity before being plunge-frozen into liquid ethane. Data were collected on a Titan Krios (FEI) operating at 300 kV, equipped with a K2 direct electron detector (Gatan). Movies were collected using Leginon (*74*) at a magnification of ×22,500, corresponding to a pixel size of 1.075 Å.

Purified HCMV Pentamer + NRP2 complex was diluted to a concentration of 0.4 mg/ml in 2 mM tris (pH 8.0), 200 mM NaCl, 2 mM CaCl_2_, 0.02% NaN_3_, and 0.01% amphipol A8-35. Protein (3 μl) was deposited on an UltrAuFoil 1.2/1.3 grid that was plasma-cleaned at 25 mA for 2 min using a Solarus plasma cleaner (Gatan). Excess liquid was blotted away for 3 s in a Vitrobot Mark IV (FEI) operating at 4°C and 100% humidity before being plunge-frozen into liquid ethane. Data were collected on a Titan Krios (FEI) operating at 300 kV, equipped with a K3 direct electron detector (Gatan). Movies were collected using SerialEM (*75*) at a magnification of ×22,500, corresponding to a pixel size of 1.073 Å.

### Cryo-EM data processing and model building

Motion correction, contrast transfer function (CTF) estimation, and nontemplated particle picking using BoxNet were performed in Warp (*76*). Extracted particles were imported into cryoSPARC (*77*) for two-dimensional (2D) classification, ab initio 3D reconstruction calculation, 3D classification, and nonuniform refinement (*78*). On the basis of the flexibility of the interface between the gH/gL and UL proteins, particle subtraction and focused refinement were also performed in cryoSPARC. Final reconstructions were sharpened with DeepEMhancer (*79*). A full description of the cryo-EM data processing workflows can be found in figs. S1, S4, and S5. Crystal structures were docked into cryo-EM density maps using Chimera (*80*) before being refined in Coot (*70*), PHENIX (*71*), and ISOLDE (*72*). Full cryo-EM data collection and refinement statistics can be found in table S2.

### Surface plasmon resonance

Purified His-tagged Pentamer was immobilized to a single flow cell of a Ni-NTA sensor in Biacore X100 (GE Healthcare) to a level of ~800 response units using HBS-P+ buffer adjusted to a pH of 8.0. Twofold serial dilutions of Fabs 1-103, 1-32, 2-18, and 2-25 were injected over both flow cells to measure binding kinetics. The sensor was doubly regenerated using 350 mM EDTA and 100 mM NaOH between cycles. Data were double reference–subtracted and fit to a 1:1 binding model using Biacore Evaluation Software (GE Healthcare).

### Biolayer interferometry

Purified monoFc-tagged NRP2 or NRP2 a2b1b2 was immobilized to anti-human capture (AHC) tips (ForteBio) in a buffer composed of 10 mM Hepes (pH 8.0), 150 mM NaCl, 0.05% Tween 20, bovine serum albumin (BSA; 1 mg/ml), and 2 mM CaCl_2_. Sensors were then dipped into wells containing purified HCMV Pentamer, ranging in concentration from 50 to 3.125 nM. Data were reference-subtracted and processed using Octet Data Analysis software v10.0 (ForteBio) with a 1:1 binding model. To evaluate the impact of calcium on Pentamer binding, the same experiment was performed using monoFc-tagged NRP2 in a buffer composed of 10 mM Hepes (pH 8.0), 150 mM NaCl, 0.05% Tween 20, BSA (1 mg/ml), and 2 mM EDTA.

To evaluate competition between Fabs and NRP2, monoFc-tagged NRP2 was immobilized to AHC tips in a buffer composed of 10 mM Hepes (pH 8.0), 150 mM NaCl, 0.05% Tween 20, BSA (1 mg/ml), and 2 mM CaCl_2_. Sensors were then dipped into wells containing a mixture of purified HCMV Pentamer at a concentration of 50 and 100 nM Fab. Data were reference-subtracted using Octet Data Analysis software v10.0.

To measure the binding kinetics of NRP2 to Pentamer in the presence of mAb 1-32, 1-32 IgG was immobilized to AHC sensors using a buffer composed of 10 mM Hepes (pH 8.0), 150 mM NaCl, 0.05% Tween 20, BSA (1 mg/ml), and 2 mM CaCl_2_. Tips with immobilized 1-32 were then dipped into wells containing 100 nM Pentamer. The 1-32–captured Pentamer was then dipped into wells containing untagged NRP2, ranging in concentration from 400 to 25 nM. Data were reference-subtracted and processed using Octet Data Analysis software v10.0 with a 1:1 binding model.

### HCMV neutralization assay

All of the Pentamer-specific antibodies used for the purposes of neutralization and inhibition assays were produced as described previously (*39*). A dengue virus–specific human IgG1 antibody (*81*) was used as isotype control. Fabs for neutralization and inhibition assays were generated by digesting IgG with papain (Sigma-Aldrich, P4762) and purifying as described previously (*82*). A standard neutralization assay with the Towne-ts15-rR, AD169rev, and 12 clinical isolates as shown in fig. S7 was performed in ARPE-19 cells using an immunostaining method (*83*). Neutralization assays in Fig. 6 and fig. S1 were performed in ARPE-19 cells using AD169rev-GFP strain, and virus infection was examined through GFP expression as described previously (*84*). For the standard neutralization assay, AD169rev-GFP (50 μl per well), generating about 100 GFP-positive cells, was incubated with 50 μl per well of serial twofold diluted IgG, Fab, or CytoGam (at indicated concentrations) at 37°C for 30 min and then added to confluent ARPE-19 cells grown in a 96-well plate. Mock-infected cells and cells infected with virus-only served as controls. For the post-attachment assay, ARPE-19 cells grown in a 96-well plate were precooled at 4°C for 10 min. AD169rev-GFP (50 μl per well) was allowed to attach to cells for 1 hour at 4°C. After removing unattached virus through a single wash using cold medium, the indicated IgG, diluted at concentrations of ~200 times of corresponding median inhibitory concentration (IC_50_), was added after culturing AD169rev-GFP–attached cells in a 37°C incubator. The antibody-containing medium was replaced with fresh medium without antibody 2 hours later. Mock-infected cells and cells infected with virus but not treated with antibodies served as controls. For the assay shown in fig. S1, AD169rev-GFP was preincubated with equal volumes of twofold serially diluted NRP2-His, NRP2-Fc, NRP2 a2b1b2–monomeric Fc, or Fc protein for 30 min before being added to confluent ARPE-19 cells grown in a 96-well plate. Mock-infected cells and cells infected with virus-only served as controls. For all above assays, triplicate wells were determined for each condition and viral infection was examined at 48 hours after infection. A C.T.L. ImmunoSpot analyzer was used to capture whole-well images of GFP expression and quantification of GFP-positive cells. The percentage of viral inhibition by the antibody and the IC_50_ of each antibody were calculated by nonlinear fit of virus inhibition % versus concentration (ng/ml) using GraphPad Prism 5 software.
