# Supplementary material for: Structural basis for HCMV Pentamer recognition by neuropilin 2 and neutralizing antibodies
Source: Sci Adv. 2022 Mar 11;8(10):eabm2546. doi: 10.1126/sciadv.abm2546 (PMC8916728; doi:10.1126/sciadv.abm2546)
Supplement: Supplementary file 1 — Figs. S1 to S10 Tables S1 and S2 [file sciadv.abm2546_sm.pdf]

Science Advances
AAAS

Supplementary Materials for

Structural basis for HCMV Pentamer recognition by neuropilin 2 and neutralizing antibodies

Daniel Wrapp, Xiaohua Ye, Zhiqiang Ku, Hang Su, Harrison G. Jones, Nianshuang Wang, Akaash K. Mishra, Daniel C. Freed, Fengsheng Li, Aimin Tang, Leike Li, Dabbu Kumar Jaijyan, Hua Zhu, Dai Wang, Tong-Ming Fu, Ningyan Zhang, Zhiqiang An*, Jason S. McLellan*

*Corresponding author. Email: zhiqiang.an@uth.tmc.edu (Z.A.); jmclellan@austin.utexas.edu (J.S.M.)

Published 11 March 2022, *Sci. Adv.* **8**, eabm2546 (2022)
DOI: 10.1126/sciadv.abm2546

This PDF file includes:

Figs. S1 to S10
Tables S1 and S2

Figure S1

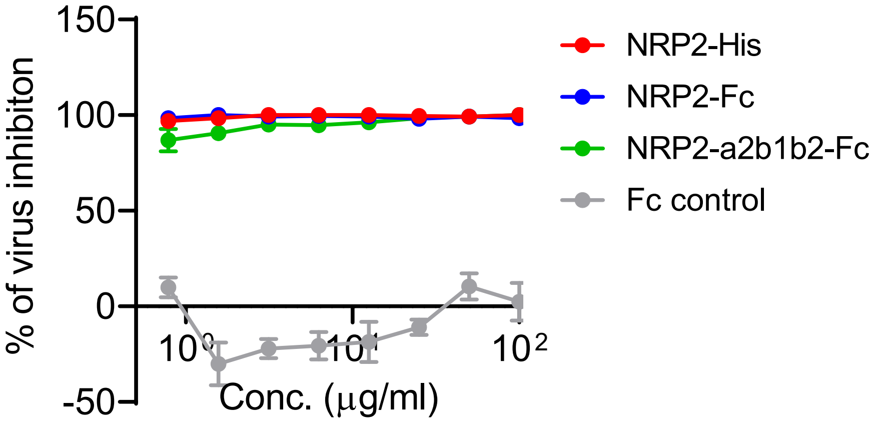

Figure S1: Pre-incubation with recombinant NRP2 proteins inhibits HCMV infection. AD169rev-GFP was pre-incubated with equal volumes of twofold serially diluted NRP2-His, NRP2-Fc, NRP2 a2b1b2-Fc or Fc protein for 30 mins before being added to confluent ARPE-19 cells. Mock-infected cells and cells infected with virus-only served as controls. Viral infection was examined at 48 h post-infection.

Figure S2

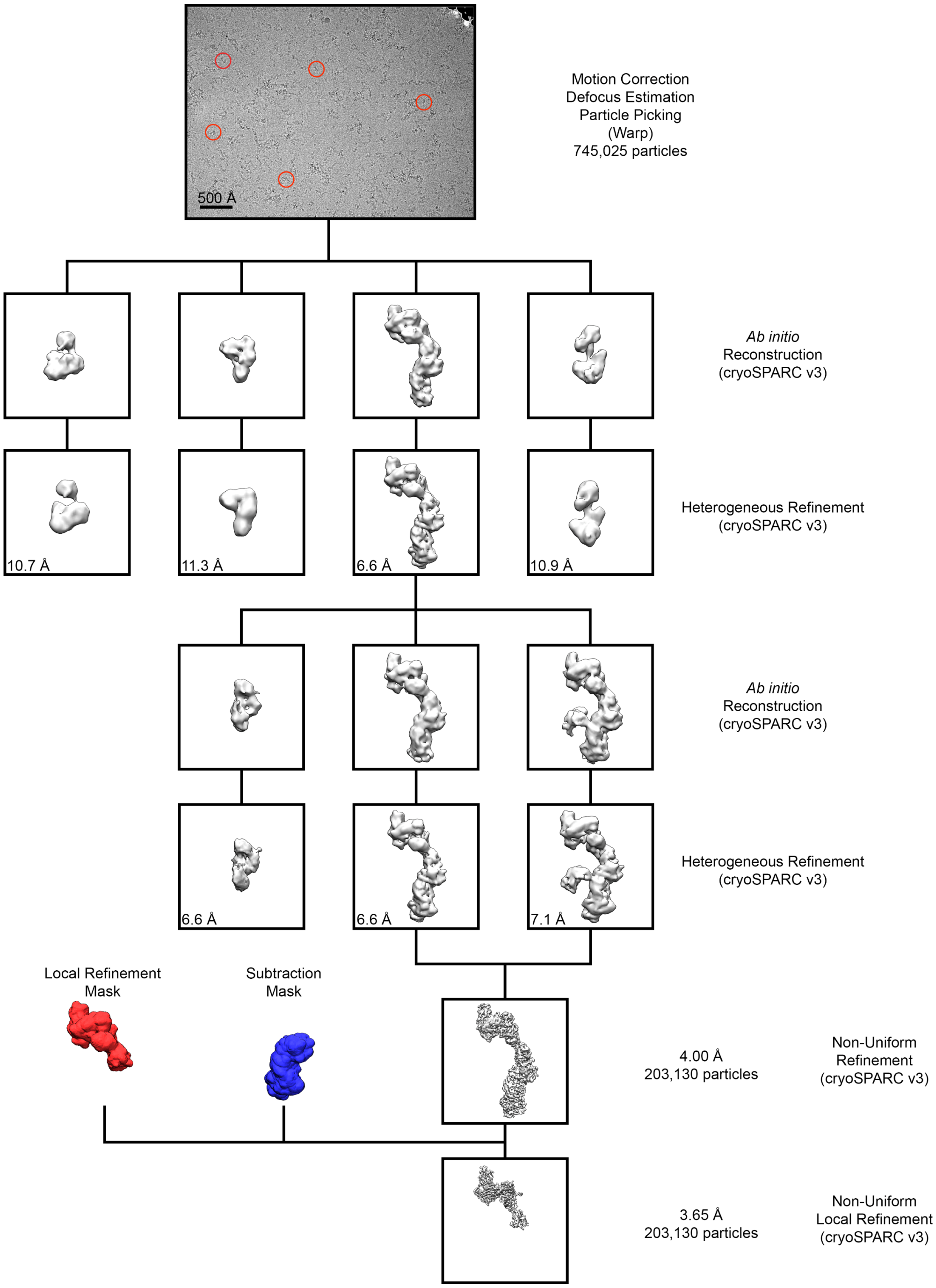

Figure S2: Pentamer + NRP2 cryo-EM data processing workflow. Red circles highlight a few of the selected particles.

Figure S3

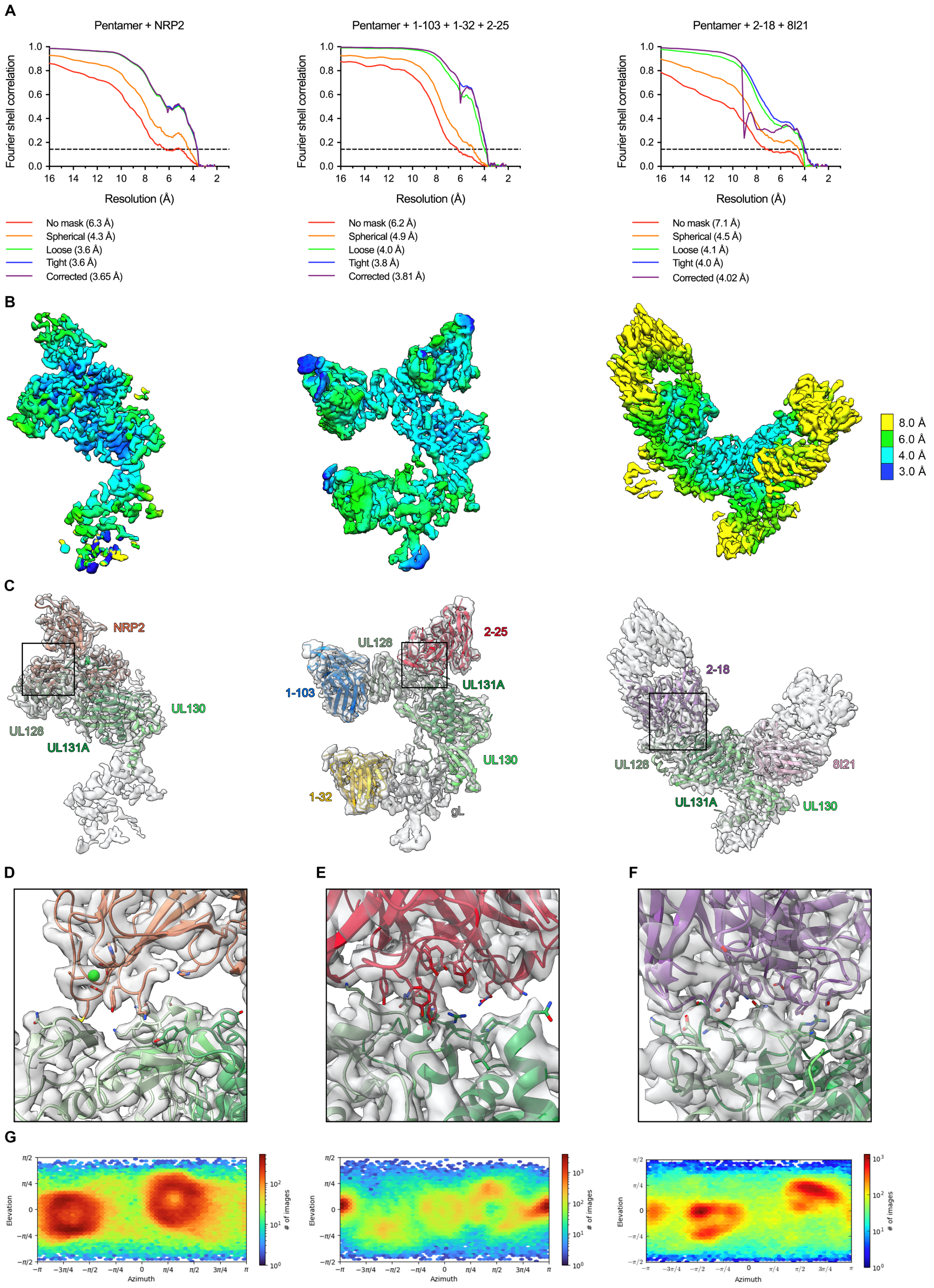
A
Pentamer + NRP2
Pentamer + 1-103 + 1-32 + 2-25
Pentamer + 2-18 + 8I21
Fourier shell correlation
Resolution (Å)
No mask (6.3 Å)
Spherical (4.3 Å)
Loose (3.6 Å)
Tight (3.6 Å)
Corrected (3.65 Å)
No mask (6.2 Å)
Spherical (4.9 Å)
Loose (4.0 Å)
Tight (3.8 Å)
Corrected (3.81 Å)
No mask (7.1 Å)
Spherical (4.5 Å)
Loose (4.1 Å)
Tight (4.0 Å)
Corrected (4.02 Å)
B
8.0 Å
6.0 Å
4.0 Å
3.0 Å
C
NRP2
UL128
UL131A
UL130
2-25
1-103
1-32
gL
2-18
8I21
D
E
F
G
Elevation
Azimuth
# of images

Figure S3: Cryo-EM structure validation. **(A)** FSC curves are shown for focused refinements of Pentamer bound by NRP2 (*left*), Pentamer bound by 1-103, 1-32 and 2-25 (*middle*) and Pentamer bound by 2-18 and 8I21 (*right*). **(B)** Cryo-EM maps from each focused refinement are shown, colored according to local resolution. **(C)** Each focused cryo-EM map is shown as a transparent surface, with the corresponding atomic models docked into the density and colored according to **Figures 2** and **4**. Boxes denote the views that are shown in panels **D-F**. (**D**) Binding interface from the NRP2-bound structure is displayed, with the cryo-EM map shown as a transparent surface and the atomic model colored according to **Figure 2**. (**E**) Binding interface from the 1-103, 1-32 and 2-25-bound structure is displayed, with the cryo-EM map shown as a transparent surface and the atomic model colored according to **Figure 4**. (**F**) Binding interface from the 2-18 and 8I21-bound structure is displayed, with the cryo-EM map shown as a transparent surface and the atomic model colored according to **Figure 4**. (**G**) Viewing Direction Distribution charts from cryoSPARC are shown for each focused refinement.

Figure S4

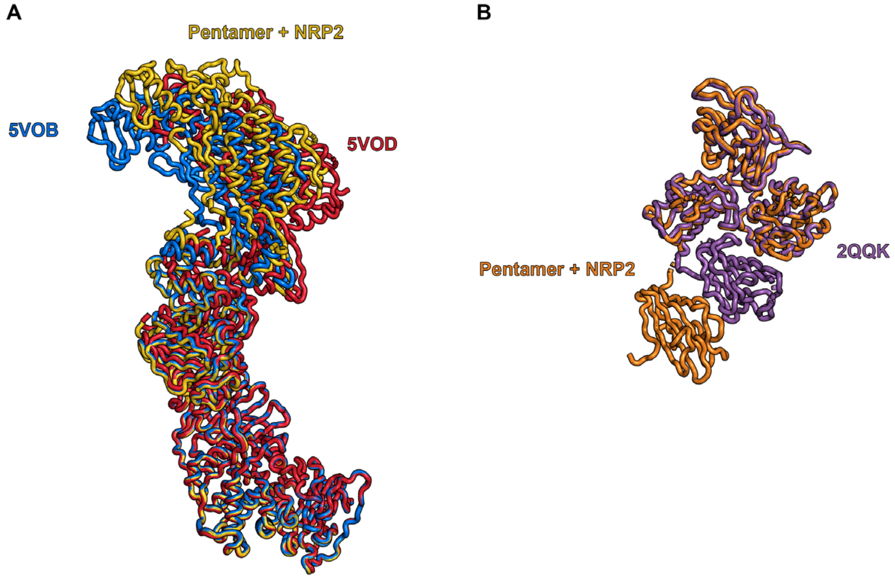

Figure S4: NRP2 binding does not alter the conformation of Pentamer. (**A**) Previously reported crystal structures of Pentamer (*45*) (PDB IDs: 5V0B and 5V0D) are aligned to the cryo-EM structure of the NRP2-bound Pentamer, based on the position of gH. 5V0B is colored blue, 5V0D is colored red and NRP2-bound Pentamer is colored yellow. (**B**) A previously reported crystal structure of NRP2 (*41*) (PDB ID: 2QQK) is aligned to the cryo-EM structure of Pentamer-bound NRP2, based on the position of the b1 domain. 2QQK is colored purple and Pentamer-bound NRP2 is colored orange.

Figure S5

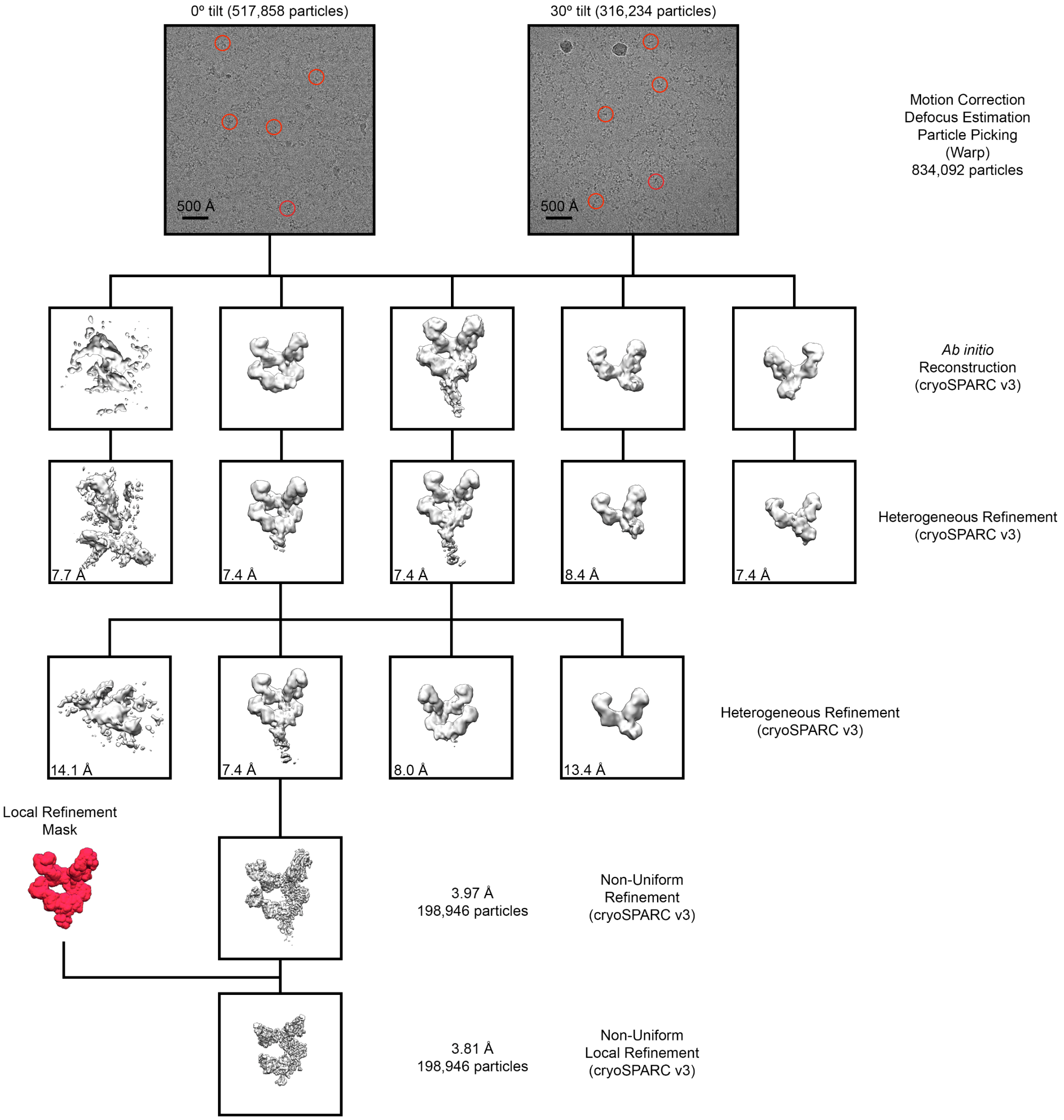

Figure S5: Pentamer + 1-103 + 1-32 + 2-25 cryo-EM data processing workflow. Red circles highlight a few of the selected particles.

Figure S6

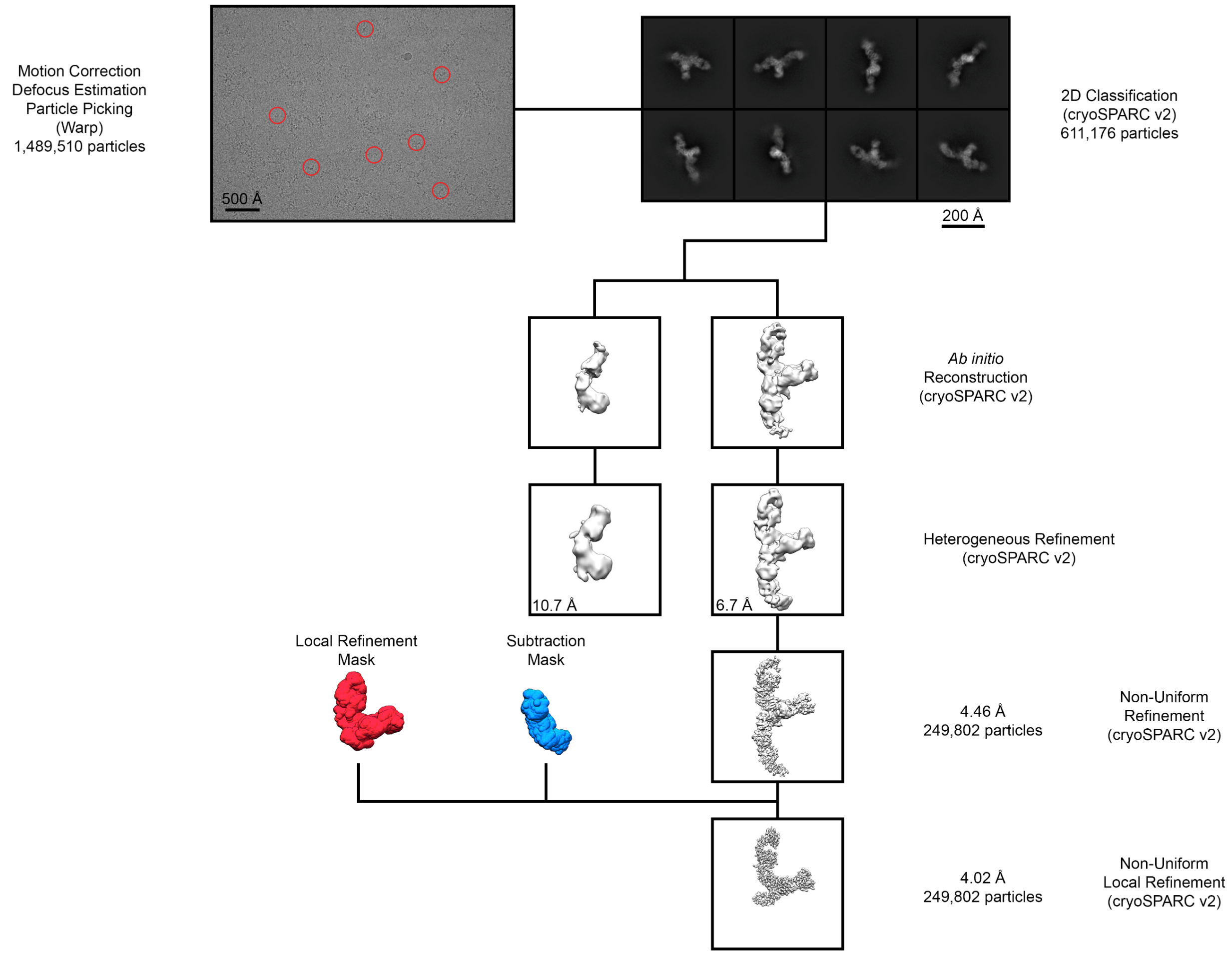

Figure S6: Pentamer + 2-18 + 8I21 cryo-EM data processing workflow. Red circles highlight a few of the selected particles.

Figure S7

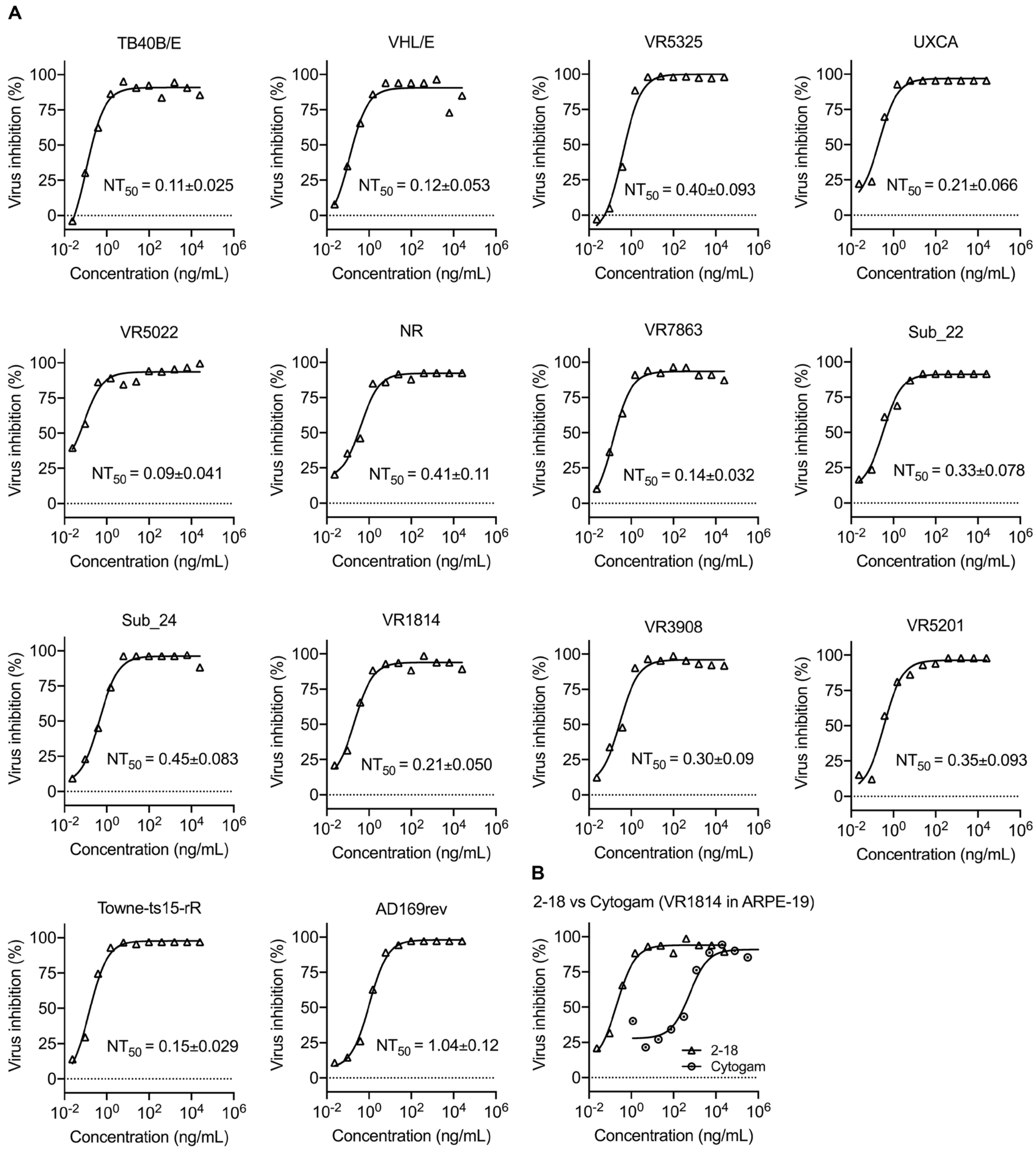

Figure S7: Neutralization activity of 2-18 against a panel of HCMV strains. (**A**) The ability of 2-18 IgG to neutralize 12 clinical HCMV isolates and two Pentamer-restored laboratory HCMV strains was evaluated using ARPE-19 cells. IC_{50} values were calculated by non-linear fit of the percentage of virus inhibition vs. IgG concentration (ng/mL) using GraphPad Prism® 5 software. (**B**) The neutralization activity of 2-18 was compared with Cytogam® against strain VR1814 in ARPE-19 cells.

Figure S8

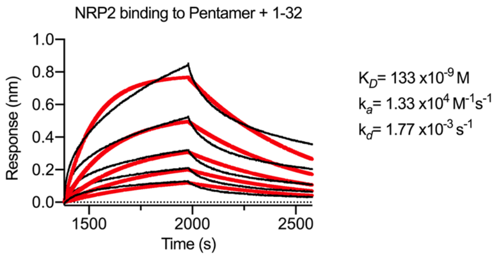

Figure S8: NRP2 binding to Pentamer is partially disrupted by the presence of 1-32. Sensorgrams are shown for an experiment in which 1-32 IgG was immobilized to a BLI sensor, then used to capture Pentamer before being dipped into NRP2. Data for the association and dissociation of NRP2 are shown as black lines and the lines of best fit of a 1:1 binding model are shown as red lines.

Figure S9

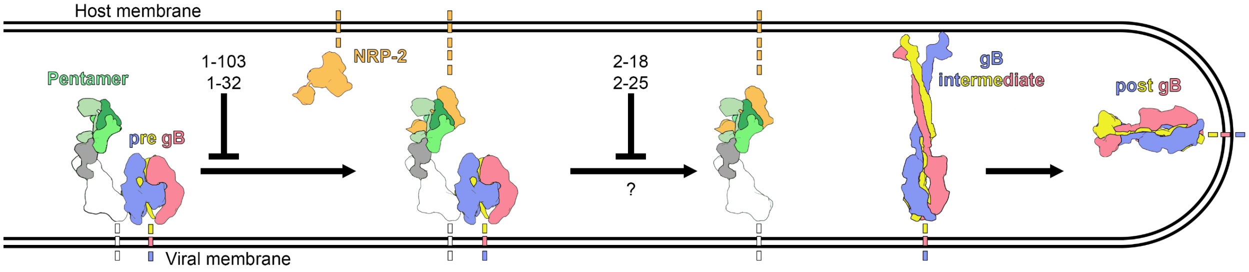

Figure S9: Pentamer-directed antibodies neutralize via distinct mechanisms. A cartoon is shown depicting the infection of an endothelial or epithelial cell by HCMV. Pentamer is colored according to **Figure 2**, NRP2 is colored orange and the three protomers of gB are colored red, blue and yellow. The stages of infection that mAbs 1-103, 1-32, 2-18 and 2-25 are predicted to disrupt are denoted. Pentamer is thought to associate with prefusion gB on the viral surface to act like the pin in a grenade, preventing the metastable prefusion state of gB from triggering to the postfusion state in absence of a host cell receptor. NRP2 binding to Pentamer is thought to induce a rigid-body motion that causes Pentamer and gB to dissociate.

Figure S10

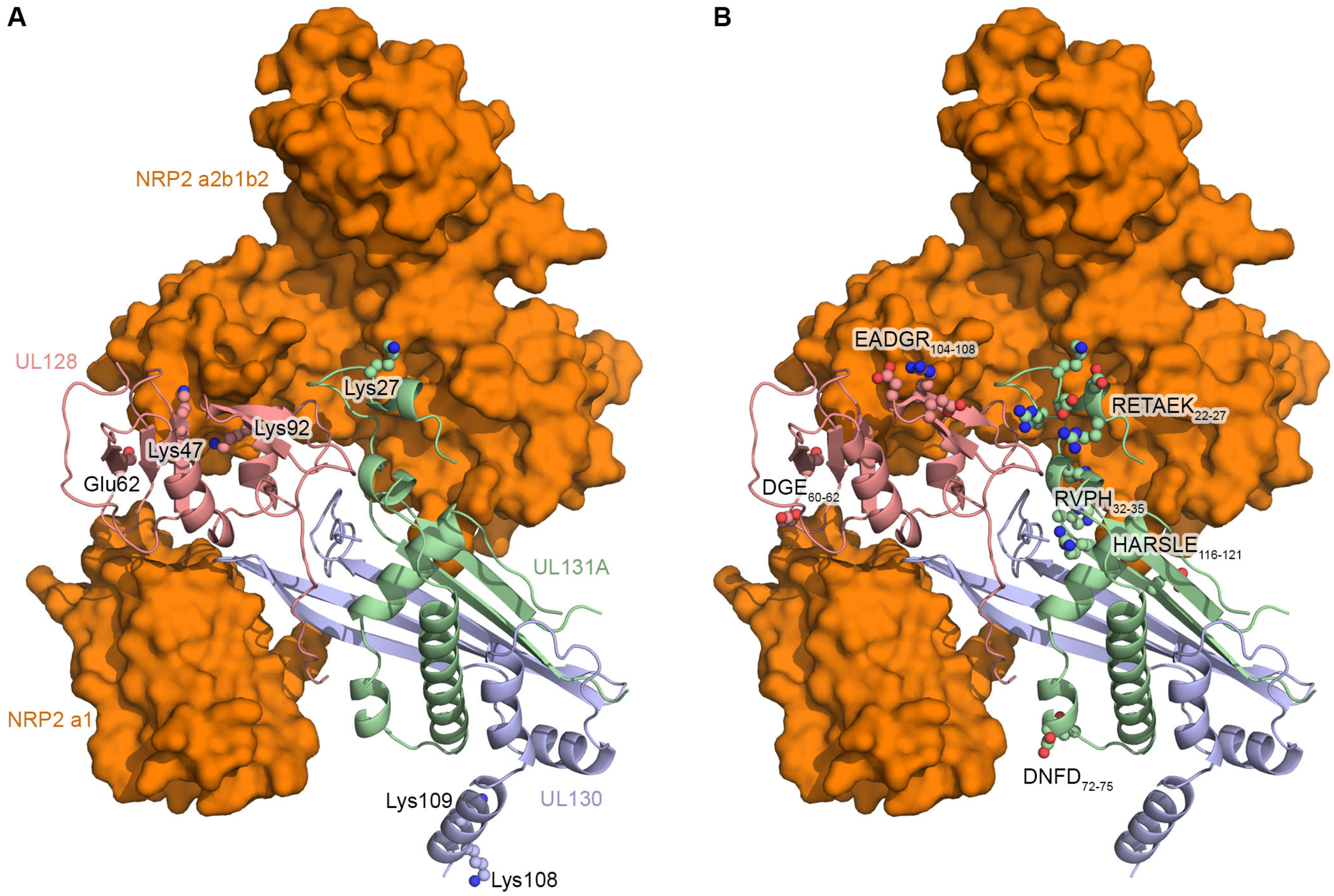

Figure S10: Previously reported NRP2-interacting residues. (**A**) Sidechains of select residues identified by chemical cross-linking and mass spectrometry in (*16*) are shown as spheres with nitrogen atoms colored blue and oxygen atoms colored red. Residues in UL128 and UL131A that were cross-linked to the a2b1b2 domains of NRP2 agree well with the cryo-EM structure reported here. Residues Lys108 and Lys109 in UL130 were found to crosslink with the a1 domain but are located about 25 Å away in the structure. This may be due to the weak affinity of the a1 domain for the UL proteins and the flexibility of a1 relative to a2b1b2. (**B**) Sidechains of select residues identified by charge cluster-to-alanine (CCTA) scanning in (*55-57*) that disrupted endothelial cell infection are displayed and colored as in (**A**). Several of the identified charged clusters in UL128 and UL131A map at or near the NRP2 interface. Charge clusters in UL130 were not located at or near NRP2 interfaces (not shown), suggesting that their effect on endothelial cell infection is due to disruption of other function(s) besides NRP2 binding.

Table S1

	1-103 Fab	1-32 Fab	2-18 Fab	2-25 Fab
**Data collection**				
Facility	APS 19-ID	APS 19-ID	APS 19-ID	APS 19-ID
Wavelength (Å)	0.979	0.979	0.979	0.979
Space group	$P2$	$P4_1\,2_1\,2$	$C2$	$P4_3\,2_1\,2$
Cell dimensions				
a, b, c (Å)	95.4, 105.2, 102.7	65.7, 65.7, 191.9	129.5, 59.2, 141.7	180.9, 180.9, 138.9
α, β, γ (°)	90.0, 91.4, 90.0	90.0, 90.0, 90.0	90.0, 91.5, 90.0	90.0, 90.0, 90.0
Resolution range (Å)	52.62-1.90 (1.93-1.90)	54.20-2.10 (2.16-2.10)	65.02-2.80 (2.95-2.80)	69.89-2.51 (2.57-2.51)
R_{merge}	0.059 (0.407)	0.174 (0.951)	0.352 (1.283)	0.187 (2.033)
CC1/2	0.996 (0.736)	0.987 (0.753)	0.851 (0.291)	0.991 (0.522)
I/σI	5.9 (1.8)	6.0 (1.5)	4.0 (1.7)	9.3 (2.0)
Completeness (%)	90.2 (91.3)	99.9 (99.8)	98.8 (96.7)	98.6 (99.9)
Redundancy	2.0 (2.0)	7.2 (6.6)	4.1 (3.8)	6.8 (7.1)
**Refinement**				
No. reflections	143,434 (14,383)	25,398 (2,476)	26,469 (2,446)	77,206 (7,680)
R_{work}/R_{free} (%)	19.1/21.7	26.8/29.7	22.1/25.5	18.5/21.9
No. non-hydrogen atoms				
Protein	12,781	3,226	6,726	9,633
Ligand/ion	0	0	0	0
Water	1,627	70	70	507
B-factors (Å^2)				
Protein	28.7	62.9	27.0	39.9
Solvent	38.8	49.7	27.1	42.9
Wilson B-factor (Å^2)	23.9	42.8	28.3	38.7
R.m.s. deviations				
Bond lengths (Å)	0.007	0.013	0.011	0.011
Bond angles (°)	1.33	1.41	1.46	1.23
Ramachandran (%)				
Favored	98.4	95.7	97.3	98.1
Allowed	1.6	4.3	2.7	1.9
Outliers	0	0	0	0
**PDB ID**	7LYV	7M1C	7KBA	7LYW

Table S1: X-ray crystallographic data collection and refinement.

Table S2

	Pentamer + NRP2	Pentamer + 2-18 + 8I21	Pentamer + 1-103 + 2-25 + 1-32
**Data collection and processing**			
Magnification (nominal)			
Voltage (kV)	300	300	300
Electron exposure ($e^-/Å^2$)	80	36	36
Defocus range (μm)	1.0-2.0	1.0-3.0	1.1-2.4
Pixel size (Å)	1.073	1.047	1.075
Symmetry imposed	n/a (C1)	n/a (C1)	n/a (C1)
Initial particles	745,025	1,489,510	834,092
Final particles	203,130	249,802	198,946
Map resolution (Å)	4.00	4.46	3.97
Focused refinement resolution (Å)	3.65	4.02	3.81
FSC threshold	0.143	0.143	0.143
**Refinement**			
Initial model(s) used (PDB ID)	5V0B, 2QQK	5V0B, 7KBA	5V0B, 7LYV, 7M1C, 7LYW
Model composition			
Protein atoms	6011	6422	9163
Protein B-factors (mean)	82.1	43.6	83.1
R.m.s. deviations			
Bond lengths (Å)	0.005	0.007	0.006
Bond angles (°)	1.14	1.38	1.38
**Validation**			
Molprobity score	1.69	2.21	1.84
Clashscore	7.47	14.42	8.91
Rotamer outliers (%)	0.61	1.58	0.89
Ramachandran plot			
Favored (%)	95.9	94.1	94.7
Allowed (%)	4.1	5.4	5.3
Outliers (%)	0.0	0.5	0.0
EMRinger score	2.84	1.21	1.80
**Data Availability**			
EMDB	23629	22788	23640
PDB	7M22	7KBB	7M30

Table S2: Cryo-EM data collection and refinement.
